# Deposition efficiency of inhaled particles (15-5000 nm) related to breathing pattern and lung function: an experimental study in healthy children and adults

**DOI:** 10.1186/s12989-017-0190-8

**Published:** 2017-04-08

**Authors:** Jenny Rissler, Anders Gudmundsson, Hanna Nicklasson, Erik Swietlicki, Per Wollmer, Jakob Löndahl

**Affiliations:** 1 Chemistry, Materials and Surfaces, SP Technical Research Institute of Sweden, Ideon Gateway, Sheelevägan 27, SE-223 70 Lund, Sweden; 2 Ergonomics and Aerosol Technology, Lund University, Box 118, SE-221 00 Lund, Sweden; 3 Clinical Physiology and Nuclear Medicine, SE-205 02 Malmö, Sweden; 4 Division of Nuclear Physics, Lund University, Box 118, SE-221 00 Lund, Sweden; 5 NanoLund, Lund University, Box 118, SE-221 00 Lund, Sweden

**Keywords:** Aerosols, Airborne particles, Respiratory tract deposition, Particle lung deposition, NanoSafety, Airway dead space, Lung dose, Inhalation, Individual variability

## Abstract

**Background:**

Exposure to airborne particles has a major impact on global health. The probability of these particles to deposit in the respiratory tract during breathing is essential for their toxic effects. Observations have shown that there is a substantial variability in deposition between subjects, not only due to respiratory diseases, but also among individuals with healthy lungs. The factors determining this variability are, however, not fully understood.

**Method:**

In this study we experimentally investigate factors that determine individual differences in the respiratory tract depositions of inhaled particles for healthy subjects at relaxed breathing. The study covers particles of diameters 15–5000 nm and includes 67 subjects aged 7–70 years. A comprehensive examination of lung function was performed for all subjects. Principal component analyses and multiple regression analyses were used to explore the relationships between subject characteristics and particle deposition.

**Results:**

A large individual variability in respiratory tract deposition efficiency was found. Individuals with high deposition of a certain particle size generally had high deposition for all particles <3500 nm. The individual variability was explained by two factors: breathing pattern, and lung structural and functional properties. The most important predictors were found to be breathing frequency and anatomical airway dead space. We also present a linear regression model describing the deposition based on four variables: tidal volume, breathing frequency, anatomical dead space and resistance of the respiratory system (the latter measured with impulse oscillometry).

**Conclusions:**

To understand why some individuals are more susceptible to airborne particles we must understand, and take into account, the individual variability in the probability of particles to deposit in the respiratory tract by considering not only breathing patterns but also adequate measures of relevant structural and functional properties.

**Electronic supplementary material:**

The online version of this article (doi:10.1186/s12989-017-0190-8) contains supplementary material, which is available to authorized users.

## Background

Exposure to airborne particulate matter impacts global health in many ways. It has toxic effects due to urban air pollution or indoor smoke from combustion sources; the transmission of infectious diseases due to bacteria (e.g., tuberculosis) or viruses (e.g., measles or SARS); and allergic diseases, where small amounts of specific substances cause problems for groups with hypersensitivity of the immune system [1, 2]. The probability of particulate matter to deposit in the respiratory tract during breathing is essential to the health effects of the particles and is also of relevance for pulmonary drug delivery. A substantial variability in deposition between subjects has been observed, not only among those with respiratory diseases, but also among individuals with healthy lungs (e.g., [3–9]). The factors determining this variability are, however, not fully understood.

The particle deposition fraction, DF, of inhaled aerosols varies by more than one order of magnitude – from less than 0.1 to almost 1 – depending on breathing pattern, structure of the respiratory tract, and particle characteristics such as size, shape and hygroscopicity [10]. Theoretical model calculations of DF provide valuable information about regional deposition and deposition in different airway generations. But models based on general assumptions are not able to describe the full complexity of the inhalation system, let alone all aspects of the biological variability between individuals (e.g., [11, 12]). Thus, models still need to be validated by experimental measurements.

The available experimental data on respiratory tract deposition of aerosol particles are fragmented and difficult to evaluate because of the lack of a common methodological standard. Typically, studies are limited to less than 10 subjects and several of the most influential only include 2–5 healthy (male) adults (e.g., [13–18]), with some exceptions (e.g., [19–21]). The information on parameters that determine inter-subject variability is very limited, partly because most measurements are made for controlled breathing over a narrow particle size interval. Experimental data on DF for children are almost completely lacking [20, 22, 23]. Furthermore, there are substantial methodological uncertainties [24]. In many studies, a medical examination of the subjects is omitted or, if included, limited to basic lung function through normal spirometry. Thus, DF can be related to breathing pattern, which usually is reported, but not to the structural and functional properties of the lung. In addition, the measurement methods are often biased by issues such as particle losses in the mouthpiece and tubing, pressure variations, failure to account for the increased humidity of the exhaled air and limitations of the available detection techniques.

In this research we used a well-characterized methodology [25–27] to measure the fully size-resolved DF. In the study we combined four experimental features: (i) a large group of subjects (67), (ii) a broad age interval (7–70 years), (iii) size-resolved particle deposition over a wide size range (15–5000 nm), and (iv) a comprehensive examination of pulmonary function. In so doing, we were able to broadly explore the most important associations between DF and breathing pattern, lung function and particle deposition efficiency.

## Method

### Study design

The study included in total 67 non-smoking participants, aged 7–67 years (sumarized in Table 1). Seven of the participants were 7–12 years old, while there were no participants in the interval 12–20 years. Excluding subjects in this age interval was motivated by the rapid development of the lungs that occurs sometime during this age with a high intra-individual variability. All subjects were subjectively healthy and no subject reported any history of disease that was expected to affect lung function or aerosol deposition. No subject reported a smoking history >1 pack a year, and all subjects were required to have lung function measurements within the normal ranges. The study was reviewed and approved by the regional ethics committee in Lund, Sweden, (dnr 2009/465) in accordance with the Declaration of Helsinki. All participants gave their written informed consent.

**Table 1:** Overview of the individuals recruited for the study, lung function, and breathing parameters

Age (y)	Males	Females	Height (cm)	Weight (kg)	V_T_ (L)	T_bc_ (min)	V_E_ (L/min)	V_Daw_ (L)	R5 (kPa · s/L)	R20 (kPa · s/L)
7–12	7		142 (±14)	40 (±13)	0.51 (±0.13)	0.063 (±0.011)	8.1 (±0.9)	0.098 (±0.030)	0.66 (±0.17)	0.49 (±0.09)
20–29	6	13	170 (±8)	64 (±12)	0.73 (±0.22)	0.100 (±0.042)	7.6 (±1.3)	0.155 (±0.045)	0.33 (±0.08)	0.31 (±0.06)
30–39	6	7	177 (±11)	73 (±13)	0.77 (±0.29)	0.102 (±0.035)	7.5 (±0.9)	0.177 (±0.079)	0.35 (±0.06)	0.33 (±0.08)
40–49	3	3	174 (±8)	73 (±13)	0.71 (±0.18)	0.085 (±0.023)	8.5 (±1.3)	0.203 (±0.065)	0.32 (±0.12)	0.30 (±0.13)
50–59	5	7	173 (±8)	82 (±13)	0.79 (±0.22)	0.104 (±0.035)	7.8 (±1.2)	0.175 (±0.053)	0.35 (±0.11)	0.29 (±0.08)
60–70	5	5	171 (±8)	79 (±11)	0.73 (±0.14)	0.103 (±0.019)	7.2 (±1.2)	0.236 (±0.065)	0.33 (±0.10)	0.25 (±0.07)
Ad. av.	25	35	173 (±9)	73 (±14)	0.75 (±0.22)	0.100 (±0.034)	7.6 (±1.2)	0.183 (±0.065)	0.34 (±0.09)	0.30 (±0.08)

Values in parentheses correspond to one standard deviation. The parameters V_T_ = tidal volume; T_bc_ = time of a breath cycle; V_E_ = minute volume ventilation rate are values recorded during the deposition measurements. V_Daw_ corresponds to anatomical airway dead space and R5 and R20 to the resistance of the respiratory system measured with impulse oscillometry at 5 Hz and 20 Hz, respectively

The size-dependent respiratory tract deposition pattern of hydrophobic particles was measured during spontaneous breathing through a mouthpiece, while sitting in a relaxed position. For each participant a comprehensive lung function investigation was performed. The particle respiratory tract deposition and lung function were measured on two separate occasions.

### Lung function measurements

Each subject went through comprehensive pulmonary function measurements. The parameters determined in the lung function tests are listed and described in more detail in the Additional file 1. Vital capacity (VC), forced expiratory volume in 1 s (FEV_1_), total lung capacity (TLC), residual volume (RV), functional residual capacity (FRC), and diffusing capacity for CO (D_L,CO_) were measured according to current guidelines [28–30] using Masterscreen Body, Viasys GmbH - Erich Jaeger, Hoechberg, Germany. Lung mechanics variables (Fres, AX, R5, R20, etc.) were studied with impulse oscillometry (IOS, Viasys GmbH - Erich Jaeger [31]). Respiratory dead space (V_Daw_) was measured by a single breath wash-out of CO_2_ (MasterScreen Capno, Viasys GmbH - Erich Jaeger) and delineated according to Wolff, Brunner [32].

In Tables 1 and 2, selected lung function data are presented together with percent of predicted lung function. The selection of the variables included in the primary statistical analysis was based on the assumed relevance for respiratory tract deposition. Variables reflecting the structure and mechanics of the lungs were thus included rather than variables reflecting gas exchange over the alveolar-capillary barrier.

**Table 2:** Measured average lung function

							% of Predicted					
Age (y)	TLC (L)	FRC (L)	FEV_1_ (L)	VC (L)	RV (L)	FEV_1_/VC	TLC (L)	FRC (L)	FEV_1_ (L)	VC (L)	RV (L)	FEV1/VC
7–12	3.5 (±0.6)	1.5 (±0.3)	1.9 (±0.6)	2.4 (±0.6)	0.95 (±0.2)	75.2 (±9.6)	106 (±9)	106 (±34)	95 (±17)	103 (±9)	119 (±27)	91 (±11)
20–29	6.3 (±1.0)	3.2 (±0.7)	3.9 (±0.6)	4.5 (±0.6)	1.6 (±0.4)	83.6 (±4.1)	108 (±9)	108 (±16)	104 (±9)	105 (±8)	109 (±23)	100 (±5)
30–39	7.3 (±1.5)	3.6 (±0.8)	4.1 (±1.1)	5.0 (±1.3)	1.9 (±0.3)	77.2 (±5.8)	113 (±8)	113 (±18)	107 (±11)	110 (±8)	107 (±21)	94 (±7)
40–49	7.2 (±1.1)	3.8 (±1.0)	3.7 (±0.6)	4.7 (±0.7)	2.3 (±0.5)	76.3 (±7.9)	115 (±14)	119 (±26)	108 (±7)	114 (±13)	119 (±28)	95 (±10)
50–59	6.7 (±1.3)	3.3 (±1.2)	3.2 (±0.5)	4.1 (±0.9)	2.3 (±0.5)	73.5 (±5.0)	110 (±14)	103 (±28)	104 (±12)	109 (±13)	112 (±19)	94 (±6)
60–70	6.3 (±1.1)	3.2 (±0.7)	2.8 (±0.7)	3.7 (±0.9)	2.3 (±0.3)	70.2 (±4.4)	104 (±10)	100 (±14)	101 (±17)	107 (±18)	104 (±11)	92 (±5)
Ad. av.	6.7 (±1.3)	3.4 (±0.9)	3.6 (±0.9)	4.7 (±1.1)	2.0 (±0.5)	77.2 (±7.0)	110 (±11)	108 (±20)	105 (±11)	108 (±12)	109 (±20)	96 (±7)

Values in parentheses correspond to one standard deviation

*TLC* total lung capacity, *FRC* functional residual capacity, *FEV*_*1*_ forced expiratory volume in 1 s, *RV* residual volume, *VC* vital capacity

### Particle deposition fraction measurements

The measurements were performed for spontaneous mouth breathing wearing a nose clip. As pointed out in earlier studies ([19] and references therein) this can, for some individuals, result in higher tidal volumes compared to natural relaxed breathing. In this study we observe higher tidal volumes and longer breath cycles than expected for fully natural breathing [33], but not far from (slightly higher) the default variables used in the multiple path particle dosimetry model (MPPD [34]). A shift in breathing pattern should not affect the conclusions on the factors that determine particle deposition. Lung function variables that are affected by body position and mouthpiece position, such as airway dead space, were measured with subjects sitting, as they were during the deposition measurements.

The deposition measurements began with a short test period (5 min.) during which the subjects became familiarized with the equipment. Thereafter, the deposition patterns were measured during two periods of 12 min each. To ensure complete mixing in the lungs and in the instrument, the first minute of measurement was discarded. The breathing patterns were recorded during the measurements and converted into BTPS (body temperature and pressure, saturated). The parameters V_T_ (tidal volume), T_bc_ (time of a breath cycle) and V_E_ (minute volume ventilation rate) presented in Table 1 were recorded during the respiratory tract deposition measurements.

The set-up used for the respiratory tract deposition measurements was a further development of a methodology used and described in several previous studies [24–26]. The main instrumental improvement compared to the previous studies was the extension of the particle size range measured, from 10 nm to 500 nm in the original instrument, to cover diameters up to 5 μm in the present study. In order to include large particles (>0.5 μm), several instrumental modifications were made. The main changes were: *i*) adding an Aerodynamic Particle Sizer (APS, model 3321, TSI®) for on-line characterization of particles with equivalent aerodynamic diameters from 700 to 5000 nm, and *ii*) redesigning the set-up to minimize particle losses in the apparatus from inertial impaction and gravitational settling as these deposition mechanisms are increasingly important for particles larger than 1 μm. The upper size limit was set by the particle losses in the set-up.

During the measurement, the inhaled and exhaled particles were sampled and characterized with two on-line instruments measuring the particle number size distribution: a Scanning Mobility Particle Sizer (SMPS) and an APS. The paths of the aerosol from the inhalation and exhalation tank to the SMPS or APS were designed to minimize particles losses, and were made as symmetric as possible to get similar losses in the flow lines when sampling from the inahlation tank or exhalation tank, illustrated in Additional file 1: Figure S2. From the difference in the inhaled and exhaled particle number size distributions, the particle size-resolved deposition fraction (DF) was determined. All the critical factors (e.g., total instrumental particle losses, mouthpiece dead space, heating to avoid condensation) for accurate respiratory tract deposition fraction measurements pointed out in a previous critical review were considered [24]. The new system is described in more detail elsewhere [27].

The SMPS classifies the particles according to their mobility diameter, equivalent to the thermodynamic diameter. In the set-up, the SMPS covers the size range of 15–500 nm. The mobility diameter (*d*_*me*_) is the equivalent diameter that has been proved to determine the respiratory tract deposition of particles of diameters <300 nm [26], explained by the fact that diffusion is the dominating deposition mechanism for these particles. Deposition by diffusion is governed by the particle diffusivity (Brownian motion) and is related to the mobility diameter as 1/*d*_*me*_ [35]. The APS classfies particles according to their aerodynamic particle diameter (*d*_*ae*_). In this system, the APS covers the diameter range ~700 nm to 5000 nm. The aerodynamic size is the most appropriate equivalent size determining the lung deposition of particles >500 nm, for which the main deposition mechanisms are impaction and gravitational settling.

The aerosol generated for the study consisted of hydrophobic particles in order to avoid particle hygroscopic growth in the lungs. To cover the whole particle size range (15–5000 nm), two different particles types were used: carnauba wax (in the size range of 15–500 nm), and manufactured spherical glass particles (in the range 500–5000 nm). The carnauba wax aerosol particles were generated by an in-house built evaporation-condensation generator and the glass particles were de-agglomerated and dispersed into air by a Vilnius Aerosol Generator (VAG, CH Technologies, Inc., Westwood, NJ). Details about the particle generation and size distributions are given elsewhere [27]. Due to the minima in the particle concentrations in the size range 350–700 nm, the steep slopes of the distributions, and the low counting efficiency of the APS for particles with a diameter below about 800 nm, the deposition data in the interval from 350 to 800 nm contained large uncertainties and were excluded. Furthermore, the respiratory tract deposition fraction was low in this size interval and thus only resulted in weak correlations in the statistical analysis and, accordingly, did not contribute further to the understanding of the individual variability in respiratory tract deposition.

### Statistical analysis

SPSS (IBM©SPSS© Statistics, v 23) was used for statistical analysis. To select a sub-set of relevant variables from the original data set, a factor analysis (principal component analysis, PCA) was performed. A PCA is a technique to reveal the interrelations between a set of variables and can be used to reduce the number of dimensions in the data. The PCA is here used to identify correlations among the independent variables. An outcome of the PCA is a number of components describing the variability in the data and the corresponding loadings (equivalent to standardized regression coefficients). The loadings represent the correlations between the variables and component. The variables investigated in the succeeding stepwise linear multivariate regression analysis were selected based on the PCA and a bivariate (Pearson’s) correlation analysis, together with knowledge about the variables and the particle deposition mechanisms. The significances presented are those at the 0.05 or 0.01 levels.

The multivariate regression analysis was first performed with the data set including only the adults. A second analysis followed including all subjects. The correlations between the lung function and breathing parameters with the size-dependent deposition fraction were evaluated by pooling deposition fractions in ten size intervals (logarithmic intervals from the measured size bins [4–5 bins per interval] of the SMPS and APS): 15–30, 30–50, 50–100, 100–200, 200–350, 850–1300, 1300–1900, 1900–2700, 2700–3500, and 3500–5000 nm. Due to the lower number concentrations and the steep slopes in the distributions in the size range 200 to 1300 nm, together with instrumental limitations, the most reliable intervals were considered to be the four lowest (covering 15–200 nm) as well as the four intervals in the size range 1300–5000 nm (see Tables 3 and 4).

**Table 3:**
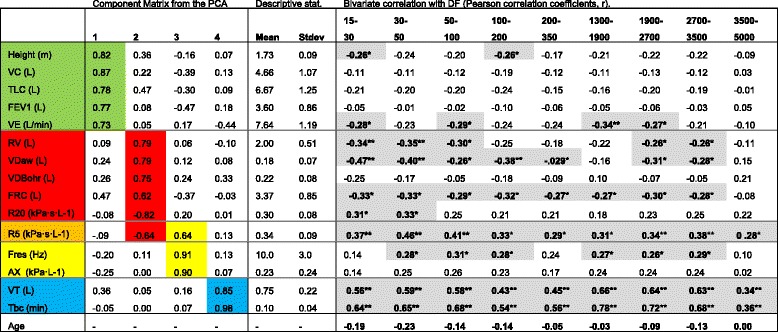
Results from the PCA, the bivariate regression analysis, and some descriptive statistics (adults)

Columns 1–4 show the results of the PCA (varimax-rotated component matrix) with PC loadings/coefficients. Color indicates the component to which each variable was mostly associated. The orange coloring of R5 indicates that this variable is equally associated to components 2 (red) and 3 (yellow). The loadings/coefficients are a measure of the correlation between the variable and the component. These columns are followed by descriptive statistics and Pearson’s coefficients from the bivariate statistical analysis of correlations between the independent variables and deposition fraction (DF). 0.05 and 0.01 significance levels are indicated by a grey background and by * and **, respectively

**Table 4:** Results from the multiple regression model of DF, based on adults only

	Unstandardized coefficients					Standardized coefficients				
Size intervals	Const.	T_bc_ [min]	V_Daw_ [L]	R5 sss [kPa · s · L^−1^]	V_T_ [L]	T_bc_	V_Daw_	R5	V_T_	R
15–30	0.60	1.07*	−0.76*	0.10**	0.14*	0.37	−0.50	0.09	0.33	0.835
30–50	0.37	1.15*	−0.67*	0.30*	0.18*	0.34	−0.38	0.23	0.35	0.824
50–100	0.19	1.69*	−0.38*	0.29*	0.09	0.51	−0.22	0.23	0.17	0.774
100–200	0.22	1.19*	−0.51*	0.10	0.04	0.46	−0.38	0.10	0.09	0.677
*200*–*350*	*0.17*	0.94*	−0.30*	*0.07*	*0.03*	*0.94*	*−0.30*	*0.07*	*0.03*	*0.642*
*850*–*1250*	*0.19*	2.47*	−0.23*	*0.10*	*−0.05*	*0.81*	*−0.14*	*0.08 *	*−0.11*	*0.750*
1300–1900	0.23	2.29*	−0.28*	0.18**	0.05	0.67	−0.16	0.14	0.10	0.815
1900–2700	0.40	1.63*	−0.62*	0.15**	0.15	0.49	−0.34	0.11	0.29	0.830
2700–3500	0.52	0.98*	−0.40*	0.19**	0.15**	0.37	−0.28	0.19	0.36	0.798
3500–5000	0.62 (0.70)	0.43* (0.34)	0.30	0.29** (0.20)	0.04 (0.07)	0.20 (0.16)	0.25	0.34 (0.24)	0.12 (0.20)	0.496

The multiple regressions include the variables T_bc_ (time of a breath cycle), V_Daw_ (anatomical dead space), R5 (airway resistance at 5 Hz), and V_T_ (tidal volume). R is the multiple correlation coefficient

For the largest particles, the analysis was made with and without V_Daw_ (latter value in parentheses). Standardized coefficients give an indication of the effect of each independent variable on the dependent variable (standardizing the variables before running the regression results in all variables being on the same scale)

Significance on a 0.05 level is indicated by*

Significance on the same level (0.05) when also including the children is indicated by**

## Results

**Fig. 1:**
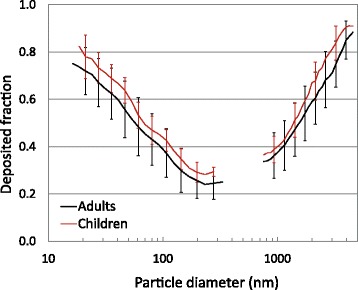
The average lung deposition fraction for adults (*solid black*) and children (*dashed red*). Error bars correspond to one standard deviation and reflect the inter-subject variation

**Fig. 2:**
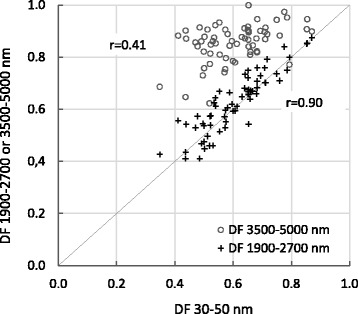
Paired DF of particles 30–50 nm with 1900–2700 or 3500–5000 nm particles. The figure illustrates that the DF of small particles correlates with the DF of larger particles up to a diameter of ~3500 nm

The average size-resolved deposition fraction found is shown in Fig. 1 for the group of adults (20–70 years) and for the children (7–12 years). The DF of the children is higher than for adults by on average 11%, but the difference is not statistically significant. Other age-dependent differences in DF within the adult group, as well as gender differences, are smaller than this and not significant (further discussed in the method paper [27]). For each individual, the deposition fraction for particles of a certain size correlate with the deposition fraction for particles of other sizes, that is, for particles with a diameter <3500 nm, as illustrated in the correlation plot in Fig. 2. Thus, individuals with a high deposition of a certain particle size tend to have a high deposition for all particles, up to 3500 nm. Typical Pearson’s correlation coefficients between the individually paired DFs of different size intervals are ~0.8–0.9. The correlations between paired DFs for particles in the size interval >3500 nm with particles <3500 are less, with Pearson’s correlation coefficients of ~0.5.

### Statistical evaluation and multivariate linear regression model

Several of the independent variables correlate with the dependent variable, DF (right part of Table 3). This is because several of the independent variables themselves are correlated. In Additional file 1 an overview of the correlation (bivariate) between the independent variables is given (Table S1). A common spirometric indicator of respiratory disease is FEV_1_. Only a few studies have investigated relationships between FEV_1_ and DF in healthy subjects and none of these found a correlation [36–38]. A significant association, however, was found in two studies including patients with COPD [36, 39]. In this study, we see no correlation with FEV_1_, as expected for a healthy group with normal spirometry at relaxed breathing for particles <5000 nm.

In order to reduce the number of variables, an exploratory factor analysis (PCA, varimax-rotated solution) was performed on the data set containing the adults. The PCA results suggest that four components explain the observed correlation among the independent variables. The corresponding loading/coefficient of each variable in each component is presented in Table 3 (adults). The table also provides the bivariate Pearson’s correlation coefficients (with significance levels of 0.01 and 0.05 indicated) between the size-dependent particle deposition fractions and the variables, as well as some descriptive statistics (average values and standard deviations).

The selection of variables used in the stepwise multivariate linear regression was based on the PCA and the bivariate correlation analysis, along with the understanding of the variables and the physical deposition mechanisms. The main variables selected to explore the deposition of particles in the respiratory tract were VC (representing principal component 1, PC1), V_Daw_ (PC2), R5 (representing both PC2 and PC3), F_res_ (PC3), and T_bc_ (C4). Other variables explored were height, V_E_, TLC (all representing PC1), RV, FRC, R20 (all representing PC2), AX (PC3) and V_T_ (PC4). More details of the variables are found in section S1 in Additional file 1.

According to the statistical analysis, the variables that best predict the deposition fraction are those belonging to principal components 2 and 4. Component 2 includes variables related to lung-intrinsic properties while component 4 includes variables related to breathing pattern. According to the stepwise multivariate linear regression analysis, these two components have significant effects on DF (*p* < 0.05) in most size intervals, except for the largest particles (>3500 nm). Variables associated with component 1 such as height, TLC, and VC did not result in significant improvement of the multivariate regression model, even if these variables partly correlate with variables in the other components and thus with DF. Component 3 did result in significant improvement of the model for some particle size intervals.

The breathing pattern is described by the two variables that were mainly associated with principal component 4: T_bc_ (time of a breath cycle) and V_T_ (tidal volume). The stepwise multivariate linear regression analysis shows that T_bc_ often better explains the deposition pattern than V_T_, but that V_T_ becomes significant, in addition to T_bc_, when looking at the deposition of the smallest particles (15–50 nm) and, when including children, also for particles >2700 nm. Even if it is not significant in all size intervals, including V_T_ as a variable in a multiple regression analysis consistently improves the correlation between the predicted and measured DF in all particle size intervals.

From the stepwise multivariate analysis, it is clear that more variables than those related to the breathing pattern (T_bc_ and V_T_) are needed to predict the inter-subject variability, especially when including the children in the analysis. For particles <3500 nm, the anatomical airway dead space (V_Daw_) is the variable related to lung-intrinsic properties that have the strongest level of explanation for the observed individual variation in DF: the larger the V_Daw_, the lower the DF. The anatomical airway dead space of the respiratory tract is composed of the portion of the airways that conducts gas to the alveoli. When excluding V_Daw_ from the analysis, FRC becomes significant, representing component 2. However, FRC does not explain the variation in DF to the same extent as V_Daw_, with the exception of large particles (1300–3500 nm) when looking at the whole group (including the children) for which FRC and V_Daw_ resulted in the same level of explanation.

Apart from the variables associated with component 2, airway resistance (partly associated with component 2 and partly with 3) measured with impulse oscillometry (IOS) was also investigated, and results in significant improvements to modelled DF for some particle sizes. The best correlation was achieved for the resistance measured at 5 Hz (R5). Including R5 in the multivariate regression analysis improved the model for all size intervals, but was significant only for 30–100 nm particles in the adult group. When including the children, R5 became significant in most size intervals.

**Fig. 3:**
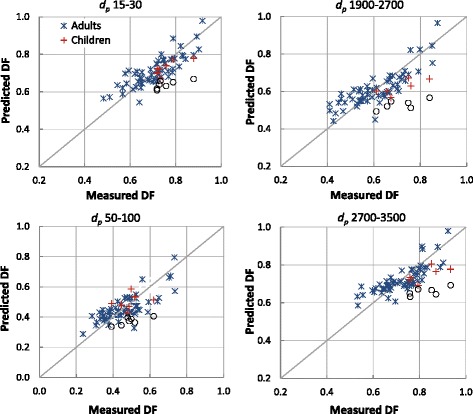
Results from the multiple regression analysis. The analysis included only adults (*blue multiplication sign*). The DF of children was also predicted (*red plus sign*) using the parameters resulting from the regression model for the adult group. To illustrate the influence of lung morphology on the DF of children, the same analysis as above was performed but only including V_T_ and T_bc_ as variables (°)

Based on the results from the stepwise multiple regression analysis, a linear multiple regression analysis was performed including the variables T_bc_, V_T_, V_Daw_ and R5 for DF in all particle size intervals. The resulting coefficients (unstandardized and standardized) are presented in Table 4. The unstandardized coefficients were used to generate the correlation plots in Fig. 3. Standardized coefficients provide an indication of the effect of each independent variable on DF, regardless of units (in contrast to the unstandardized coefficients). As can be observed from the standardized coefficients in Table 4, the variable explaining most of the variability in DF, for most size intervals, was T_bc_; however, the airway dead space (V_Daw_) was nearly as important. In the analysis, only adults were included; however, the model also applied relatively well to children, as illustrated in Fig. 3. Excluding the children from the main analysis was done to avoid effects related to differences only between the two groups (adults and children). To illustrate the influence of lung morphology on DF, the predicted DF based on a multiple regression model including only T_bc_ and V_Daw_ is also shown in Fig. 3. A similar analysis including all subjects (i.e., adults and children) is presented in Additional file 1: Table S2.

For particles >3500 nm, the significant correlation between DF and airway dead space disappears, while the significant correlation between DF and T_bc_ remains. R5 is significant when including the data set of the children. However, replacing R5 with R20 in this size interval results in a slightly stronger correlation with DF, which is not the case for DF of the smaller particles.

## Discussion

Overall, in the sub-micrometer particle size range the measured deposition fractions reported in this study are in agreement with previous studies where respiratory tract deposition has been measured for healthy subjects during spontaneous breathing of hydrophobic particles [4, 6, 9, 19, 26, 37, 39–41]. For particles larger than 1 μm, the DF values obtained are 0.2–0.3 higher than previously reported in two studies by Heyder et al. and Bennett et al. [6, 19], but lower than those measured by Montoya et al. [42] for ambient aerosols and in a similar range as the data reported by Giacomelli-Maltoni, Melandri [43] and Chan, Lippmann [3]. The DFs measured are not fully intercomparable since the breathing patterns and lung physiology of the subjects differ. This is further discussed in the method-paper [27].

Individuals with a high deposition of a certain particle size had a high deposition for all particles, up to 3500 nm. That is despite the fact that the deposition of particles with different sizes is governed by different mechanisms: typically diffusion for particles <500 nm, sedimentation for particles >500 nm, and impaction for particles >2 μm (diameters given are rough estimates and depends on flow and tube dimensions). In the peripheral airways, the dominating deposition mechanisms are diffusion and sedimentation (or gravitational settling). Diffusion and sedimentation are affected in a similar way by *i*) flow, *ii*) residence time, and *iii*) dimensions of the airways. These facts likely explain the high correlation between paired DFs of different sizes of particles <3500 nm. In the upper and more central parts of the lungs, where the air velocity is higher, inertial impaction plays a more important role. Thus, impaction is governed by other lung-intrinsic properties than sedimentation and diffusion. Contrary to diffusion and sedimentation, impaction increases with increasing flow rates. The decrease in the paired correlation between the DF for particles >3500 nm implies that impaction becomes more dominant for particles in this size interval, even at the low flow rates during normal relaxed breathing.

### Breathing variables (PC 4)

The correlation between DF and T_bc_ is explained by diffusion and sedimentation being deposition mechanisms both of which depend on the particle residence time. This has been reported earlier. Heyder et al. [44, 45] state that for both diffusion and sedimentation, the number of particles deposited in the airway dead space is negligible compared to the number deposited in the peripheral airways (for particles <2 μm). Consistent with this, Rudolf et al. [6, 17, 46] suggest that the pulmonary DF is explained to a large extent by: (*i*) the residence time of the air in the alveoli, and (ii) the fraction of the tidal volume that reaches the alveolar region (V_TA_/V_T_). These two factors were investigated by Shiller et al. [17] by altering the tidal volume in a homogeneous group of male adults (*n* = 4).

Our observations from the statistical analysis of the relative influence of V_T_ and T_bc_ are in line with Bennet et al.’s [19], who found that breathing frequency (∝1/T_bc_) best predicts the DF. They are also in line with the observations of Schiller et al. [17], who reported that for the smallest particles, V_T_ becomes more important for describing the variability in DF. Kim and Jaques [7] concluded that T_bc_ and V_T_ are equally influential on deposition for the submicron particles in the size interval 40–100 nm, while in our study the size interval was somewhat lower, 15–50 nm. For particles in the range 2700–3500 nm, though, V_T_ and T_bc_ are also equally influential. This is the size interval where deposition by sedimentation in the peripheral airways is expected to peak (at normal breathing).

For a flow-through system, such as a tube, the residence time is linearly proportional to the flow rate. However, a flow-through system is not representative for particles deposited in the peripheral lung, and accordingly the multivariate regression analysis shows that it is T_bc_ and V_T_ that determine the deposition rather than the minute volume ventilation rate (V_E_). This has been reported in several previous studies (e.g., [7, 19, 47]). For particles deposited by impaction in the upper and central parts of the lungs, the deposition more resembles that in a flow-through system, and the deposition fraction is expected to be flow dependent.

### Airway anatomy (PC 2)

Our data shows that there is a clear correlation between not only to the breathing variables associated with PC4 but also between some of the intrinsic properties of the lungs associated with PC2. In the descriptive model suggested by Kim et al. [7], only variables describing the breathing pattern was included and no lung-intrinsic properties. The study was performed on young healthy subjects following a predetermined breathing pattern with particles in the 40–100 nm range. When applying their model to the breathing parameters of the subjects of our study, it became evident that more variables were needed to explain the observed inter-subject variability in DF. The study by Kim et al., similar to many previous studies, measured the deposition fraction for a relatively homogeneous group of young healthy adults. This likely explains why in the present study, in contrast to Kim et al., we found that lung-intrinsic properties are needed to explain the individual variability in DF. This was also noted by Finlay and Martin [48] who further developed the equations suggested by Kim et al. to include FRC. Bennett et al. [19] studied the DF during controlled spontaneous breathing for a large group of people in a wide age span (18–80 years), using a monodisperse aerosol of 2 μm particles (optical equivalent diameter). They also found that the intra-subject correlations in the DF were explained by the intrinsic characteristics of the individuals, such as lung morphology. That study showed that apart from the breathing variables, the specific airway resistance (measured by body plethysmography) together with effective airspace diameter (EAD), were the best predictors of DF. EAD is a measure of the size of the airways, measured by the deposition of airborne particles during a breath hold, according to the principle described by Heyder [49]. V_Daw_ was not measured. We used spontaneous breathing in our study. The effects of lung morphology could have been better isolated using a controlled breathing pattern, which, on the other hand, would not fully reflect spontaneous breathing.

The airway dead space, V_Daw_, is the variable related to the lung-intrinsic properties that best describe DF: the smaller the V_Daw_, the higher the DF. V_Daw_ represents the anatomical dead space of the respiratory tract and is composed of the conducting airways (i.e., the portion of the airways that conducts gas to the alveoli from the mouth down to the respiratory bronchioles). About half of the V_Daw_ is in the distal airways, below generation 10 [50]. When removing V_Daw_ from the stepwise multiple regression analysis, FRC steps in and becomes significant. FRC and V_Daw_ are correlated since a small part of the FRC is composed of V_Daw,_, and possibly also since they both may reflect the general size of the respiratory tract.

In a few previous studies (e.g., [34, 48, 51]), FRC was used to account for some aspects of respiratory tract geometry. Finlay and Martin [48] proposed that further variability in DF can be explained by differences in the conducting airways. Also in the early studies by Rudolf et al. [17, 45, 46], V_Daw_ was suggested as a variable explaining DF.

The correlation between DF and airway dead space could at first be misinterpreted as a small dead space (and thus smaller dimensions of the airways) leading to higher deposition in the conducting airways. Drawing the parallel to particle losses in a tube, the losses by diffusion and sedimentation would increase with decreasing radial dimensions of the tube – if the air velocity was kept constant. However, if the air volume flow is kept constant when decreasing the dead space (and thus tube radius), the air velocity would increase, possibly leading to shorter residence times and lower deposition. To further investigate this, calculations were made of particle losses by diffusion and sedimentation in a horizontal tube. More details about these calculations are found in section S3 in Additional file 1. The calculations were performed assuming both laminar and turbulent flow and show that reducing the radius of a tube results in lower, or similar, particle depositions. Thus, the observed effect of increasing DF with decreasing V_Daw_ cannot be explained by increased deposition in the conducting airways.

As mentioned in the discussion regarding V_T_, the studies by Rudolf et al. suggest that the deposition is partly determined by the fraction of the tidal volume that reaches the lung periphery (V_TA_/V_T_), which may be also be expressed as a function of V_Daw_ as 1-V_Daw_/V_T_. Thus, a smaller airway dead space results in a larger fraction of V_T_ entering the lung periphery, which may increase the particle deposition there. This is consistent with our observations of decreasing DF with increasing V_Daw_.

In addition, the average residence time in the lung periphery, T_A_, is also proportional to V_Daw_ (with a large V_Daw_, the T_A_ becomes shorter). If making the simple assumption of a constant flow rate (square wave breathing pattern), T_A_ can be expressed as (T_bc_-T_Daw_)/2 (where T_Daw_ is the total time the air spends in V_Daw_), or (T_bc_-V_Daw_/V_E_)/2 (where V_E_ = V_T_/T_bc_). Thus, a large V_Daw_ leads to a shorter T_A_, which in turn results in a lower deposition by diffusion and sedimentation in the lung periphery. This is also consistent with our observations._._

Another possible parameter affecting the particle deposition in the peripheral airways is the dimensions of the peripheral airspaces [49]. In healthy subjects, one assumption is that the alveolar distances are reflected by the alveolar volume (V_A_). We know this is not true for people with lung disease (e.g., pulmonary emphysema resulting in structural changes in the alveoli). At the beginning of a breath, V_A_ can be estimated as FRC-V_Daw_.

To evaluate the variables T_A_, V_A_ and, V_TA_/V_T_, these were added in the stepwise multiple linear regression analysis. The analysis was carried out both for the adult group separately, and for the whole group including the children. For both groups, and for all sizes except the smallest and the largest size fraction, the best predictors were T_A_ and V_A_, replacing V_Daw_ and T_bc_ in the stepwise multiple regression analysis. However, the improvement in the correlation coefficient compared to using the original variables was minor. Also for the smallest particles (15-30 nm) T_A_ and V_A_ were significant, but V_TA_/V_T_ was stepping in with an even higher level of explanation, while for particles >2.7 μm only R5 was significant. In the new analysis R5 was no longer significant for the smaller particles, as it was in the previous analysis when looking at the whole group.

The correlation between DF and R5 is not straight forward to interpret. R5 is considered to reflect the total resistance of the respiratory system, whereas the resistance calculated at higher frequencies is considered to reflect proximal airways [52] (see Additional file 1). In healthy subjects, as in this study, the resistance of the respiratory system is low. The diameter of the airways is inversely related to their resistance, and the association between DF and R5 could therefore be a reflection of individual differences in airway dimensions in healthy subjects. The fact that R5 showed a closer correlation to DF than to R20 may indicate that the properties of the small airways have a larger impact on deposition than those of larger airways. We did not, however, find any correlation between DF and the difference between R5 and R20, which is often considered to reflect the resistance of small airways. In normal subjects, the difference between R5 and R20 is very small [52], and there may be too much noise in the data to see such correlation. Nonetheless, the relationship between resistance and DF must be interpreted cautiously. From the PCA, it appears that FRC also captures a part of PC3, and replacing R5 with FRC in the multiple regression analysis did result in nearly as high correlations.

Thus, we found that the variables reflecting the dimensions of the lower respiratory tract explain most of the individual variability in DF for particles <3500 nm, consistent with diffusion and sedimentation predominantly occurring in the peripheral airways. This was not the case for particles >3500 nm and the results indicate that impaction comes into account and thus, that DF for this size fraction is determined also by other lung-intrinsic properties than DF for particles <3500 nm. These conclusions hold for healthy subjects. For subjects with diseases resulting in structural changes or altered breathing patterns, other lung function variables may better reflect the particle lung deposition.

### Particle deposition in the airways of children

Exposure to airborne particles may pose different risks to different sub-populations, and children have been identified as one of the most sensitive groups. Efforts have been made to understand age-dependent deposition in the lungs of children (e.g., [20, 22, 51, 53]). Some of these studies have reported that one difficulty in doing so is the lack of adequate information on lung geometry during growth.

The DF for children during spontaneous breathing has not previously been investigated together with comprehensive pulmonary function measurements. It should, however, be pointed out that this study only includes 7 children. We obtain a slightly higher DF in children than for adults (11%), but the difference was not significant. Schiller-Scotland et al. [23] found that the DF for the children was 50% higher compared to adults, while Bennett, Zeman [54] did not see any significant difference. Bennett and Zeman explained the large difference between the two groups observed by Shiller-Scotland et al. as an effect of the mouthpiece, resulting in children increasing their tidal volume compared to normal relaxed breathing. The set-up may also affect breathing pattern due to the resistance of the instrument and thus, different set-ups can affect breathing pattern differently.

The difference in the deposition rate (deposited particles per time unit, i.e., taking into account the minute volume ventilation rate) between children and adults was slightly larger than in the deposition fraction, with a ~20% higher rate for the children compared to the adults. That was when sitting in a relaxed position. The real difference in deposition rate, and thus in deposited dose, is expected to be higher due to the generally higher activity level, and thus breathing volume, of children. Furthermore, the difference between adults and children becomes larger when normalizing the deposition rates with lung surface area or body mass [27], as was also reported by Bennett, Zeman [54].

As discussed earlier, and shown in Fig. 3, the DF for the children is nicely predicted with the same linear regression model as for the adults. In the study by Bennett, Zeman [54], the best predictors of the lung deposition for adults were T_bc_ and the mean effective airspace diameter (MEAD),^1^ while for children, V_T_ was a better predictor than T_bc_. We see the same trend in our data: that V_T_ becomes more important when including the children in the analysis (for particles <3500 nm). However, T_bc_ is still a better predictor, or as good as V_T_.

With the exception of children, another group expected to be sensitive to exposure to airborne particles are individuals with diseased lungs. Lung deposition data for this group are largely lacking, especially for sub-micrometer sized particles that dominate particulate air pollution by number.

## Conclusions

To understand why some individuals are more susceptible to airborne particles than others, we must take into account the individual variability in the probability of particles to deposit in the lungs. In this study we investigate factors that determine individual variability in the lung deposition of airborne particles, for healthy subjects, during relaxed breathing. We find a large individual variability in respiratory tract deposition efficiency where subjects with high deposition of a certain particle size in general have a high deposition for all particles – up to particles of a diameter of 3500 nm. The individual variability is explained by two factors: breathing pattern and lung-intrinsic properties. The most important predictors are found to be time of a breath cycle (the inverse of breathing frequency) and anatomical airway dead space, followed by tidal volume and resistance of the respiratory system (R5), measured by impulse oscillometry. We found support for the hypothesis that pulmonary particle deposition to a large extent is determined by: (*i*) the residence time of the air in the peripheral airways, and (*ii*) the fraction of the tidal volume that reaches the peripheral airways. This hypothesis explains the correlation of T_bc_ (time of a breath cycle) and T_v_ (tidal volume) with DF. The fraction of the tidal volume reaching the peripheral lungs is, apart from tidal volume, also influenced by the anatomical dead space (V_Daw_), possibly explaining one part of the (anti) correlation between DF and V_Daw_. Furthermore, the anatomical dead space affects the particle residence time in the peripheral lungs. R5 is the least influential variable of the four mentioned, and thus the most uncertain. R5 is assumed to generally reflect the dimensions of the airways, as is FRC.

## Additional file

Additional file 1: The Additional file 1 contains (i) more details on the measured lung function parameters and (ii) supplemental tables and figures: A schematic illustration of the set-up, a table presenting the bivariate correlations between selected variables, a supplemental table and figure corresponding to Table 4 and Fig. 3 in the main article including also children, and correlation plots. Finally, a table over selected lung function data and deposition fractions for all individuals is given. The Supplemental Material also contains (iii) more details on the calculation of particle losses in a tube. (DOCX 405 kb)

## Abbreviations

APS: Aerodynamic Particle Sizer
AX: Reactance area. The integrated respiratory reactance between 5 Hz and F_res_ (see Additional file 1)
BTPS: Body temperature and pressure, saturated
*d*_*ae*_: Aerodynamic particle diameter
DF: Deposition fraction. The term refers to the mean probability of an inspired particle being deposited in the respiratory tract (no bounce of particles)
*d*_*me*_: Mobility diameter
FEV_1_: Forced expiratory volume in 1 second (see Additional file 1)
FRC: Functional residual capacity (see Additional file 1)
F_res_: Resonant frequency (see Additional file 1)
IOS: Impulse oscillometry
PC: Principal component
PCA: Principal component analysis
R20: Resistance of the respiratory system measured with impulse oscillometry at 20 Hz. R20 reflects the central airways more than does R5 (see Additional file 1)
R5: Resistance of the respiratory system measured with impulse oscillometry at 5 Hz. R5 mainly reflects the resistance of conducting airways (see Additional file 1)
RV: Residual volume (see Additional file 1)
SMPS: Scanning Mobility Particle Sizer
T_A_: Alveolar residence time for the fraction of the tidal volume reaching the alveolar region (V_TA_)
T_bc_: Time of a breath cycle
T_Daw_: Residence time in airway dead space
TDF: Total deposition fraction of particles of a certain distribution
TLC: Total lung capacity (see Additional file 1)
V_A_: Volume of the pulmonary region (alveoli)
VC: Vital capacity
V_Daw_: Anatomical airway dead space (the volume of the conducting airways), or more specifically the Wolff dead space (see Additional file 1)
V_DBohr_: Bohr’s dead space (See Additional file 1)
V_E_: Minute volume ventilation rate
V_T_: Tidal volume (according to BPTS conditions
V_TA_: Volume of the tidal volume that passes the conducting airways to reach the alveolar region to take part in the gas-exchange

## Footnotes

1 MEADs were determined by analysis of total exhaled aerosol recovery following inspiratory capacity breaths of an aerosol with breath holds at total lung capacity (TLC) for 0 to 10 s of 1 μm particles.
