# Supplementary material for: Deposition efficiency of inhaled particles (15-5000 nm) related to breathing pattern and lung function: an experimental study in healthy children and adults
Source: Part Fibre Toxicol. 2017 Apr 8;14:10. doi: 10.1186/s12989-017-0190-8 (PMC5385003; doi:10.1186/s12989-017-0190-8)
Supplement: Additional file 1: — The Additional file 1 contains (i) more details on the measured lung function parameters and (ii) supplemental tables and figures: A schematic illustration of the set-up, a table presenting the bivariate correlations between selected variables, a supplemental table and figure corresponding to Table 4 and Fig. 3 in the main article including also children, and correlation plots. Finally, a table over selected lung function data and deposition fractions for all individuals is given. The Supplemental Material also contains (iii) more details on the calculation of particle losses in a tube. (DOCX 405 kb) [file 12989_2017_190_MOESM1_ESM.docx]

**Additional file 1**

## Deposition efficiency of inhaled particles (15-5000 nm) related to breathing pattern and lung function: an experimental study in healthy children and adults

# Jenny Rissler, Anders Gudmundsson, Hanna Nicklasson, Erik Swietlicki, Per Wollmer, Jakob Löndahl

**Table of Contents**

# S1 Measured lung function parameters

Figure S1

S2 Supplemental figures and tables

Figure S2

Figure S3

Figure S4

Table S1

Table S2

Table S3

S3 Particle losses in a tube

References

# S1 Measured lung function parameters

**Figure S1.** Illustrating a few of the different lung volumes described below.

**VC (vital capacity)** [L]

The maximum air volume that can be inspired/expired after full expiration/inspiration. VC was measured according to current guidelines [1-3] using Masterscreen Body, Viasys GmbH - Erich Jaeger, Hoechberg, Germany.

**TLC (total lung capacity)** [L]
The volume of air in the lungs after a maximal inspiration: the sum of the vital capacity (VC) and the residual volume (RV).

**RV (residual volume)** [L]
The air volume remaining in the lungs after a maximal expiration. This volume cannot be exhaled. RV was measured according to current guidelines [1-3] using Masterscreen Body, Viasys GmbH - Erich Jaeger, Hoechberg, Germany.

**FRC (functional residual capacity)** [L]
The volume remaining in the lungs after a normal exhalation. FRC was measured according to current guidelines [1-3] using Masterscreen Body, Viasys GmbH - Erich Jaeger, Hoechberg, Germany.

**FEV_1_ (forced expiratory volume in 1 second)** [L]

The volume exhaled during the first second of a forced expiration after a full inspiration. FEV_1_ was measured according to current guidelines [1-3] using Masterscreen Body, Viasys GmbH - Erich Jaeger, Hoechberg, Germany.

**FEV_1_/VC**
Ratio used as an indicator of airflow limitation, which occurs in obstructive airways disease and emphysema.

**IOS (impulse oscillometry)**
Impulse oscillometry (IOS, Viasys GmbH - Erich Jaeger [4]) provides a measure of the impedance (Z) of the respiratory system. A sound wave is sent into the airways while the patient is breathing normally at rest through a mouthpiece. The sound wave consists of a number of frequencies travelling superimposed upon the normal tidal breathing of the patient. Pressure and flow are measured at the airway opening and their ratio analyzed by fast Fourier transform to yield the impedance of the respiratory system, Z. Z is the sum of all forces opposing the pressure impulses: the sum of the respiratory resistance (R) and the respiratory reactance (X). R at specific frequencies is denoted R5 (for 5 Hz), R10, R20, etc. Correspondingly, reactance is denoted X5, X10, X20, etc.

In principle, the resistance at 5 Hz (R5) reflects the resistance of the entire respiratory system, including the conducting airways, the lung parenchyma and the chest wall. High oscillation frequencies (>20 Hz) are damped out in large and medium-sized airways and therefore provide little information about the peripheral airways. The difference between R5 and R20 is therefore often taken to represent the resistance of the peripheral airways.

It is well known that resistance as measured by IOS has no close correlation to FEV_1_. Resistance has a closer relation to symptoms than FEV_1_ both in subjects with chronic obstructive pulmonary disease and in asthmatics challenged with methacholine. There is also a consistent relationship between resistance and the clinical severity of chronic obstructive pulmonary disease [5].

The reactance, X, is the imaginary part of the impedance, Z. Reactance was not included in the analysis.

**F_res_ (resonant frequency)** [Hz] The oscillatory frequency at which reactance, X, is zero (see IOS above for more info). At this frequency the capacitative and inertive pressure components of X are equal. F_res_ marks the transition from capacitative dominance to inertive dominance and helps categorize frequencies as high or low. F_res_ also gives the frequency for which the impedance, Z, is only dependent on the resistance, R, of the airways. F_res_ is normally approximately 6-11 Hz. Usually F_res_ tends to be higher for children and to decrease with age. For both restrictive and obstructive diseases, F_res_ increases.

**AX (reactance area**) [kPa·s·L^-1^]
The integrated respiratory reactance between 5 Hz and F_res_ (see IOS above for more info).

**V_T_ (average tidal volume)** [L]

The average of the inhaled and exhaled volume at rest. The V_T_ presented in this article is those recorded during the lung deposition measurements, converted to BTPS.

**V_Daw_ (airway dead space)** [L]
The airway dead space was measured according to the principle of Wolff and Brunner [6]. The V_Daw_ is based on the differentiation of phase II of a single breath CO_2_ diagram and does not include the phase III in the single breath CO_2_ diagram [7]. V_Daw_ is the volume of the non-mixing expirate and is often considered as an adequate description of the conducting airways only (anatomical dead space), excluding the alveolar dead space (functional dead space). Non-mixing here includes both convective mixing and mixing by diffusion. In that sense it is the smallest estimate of the anatomical dead space.

The values used in the study are the average over four smooth breaths.

**V_DBohr_ (Bohr’s dead space)** [L]

Bohr’s dead space reflects the physiological dead space (anatomical and alveolar) and is based on the assumption that all expired CO_2_ comes from alveolar gas and estimated by the equations and description found in Kars et al. [8] (includes phases I-III of the breath [7]). Bohr’s dead space is influenced by the alveolar dead space and partly reflects ventilation-perfusion relationships in the lungs, which is not the case for Wolff dead space (equivalent to airway dead space as described above). Thus, V_Daw_ was used as the main expression of anatomical dead space.

The values used in the study are the average over four smooth breaths.

**S2 Supplemental tables and figures**

**
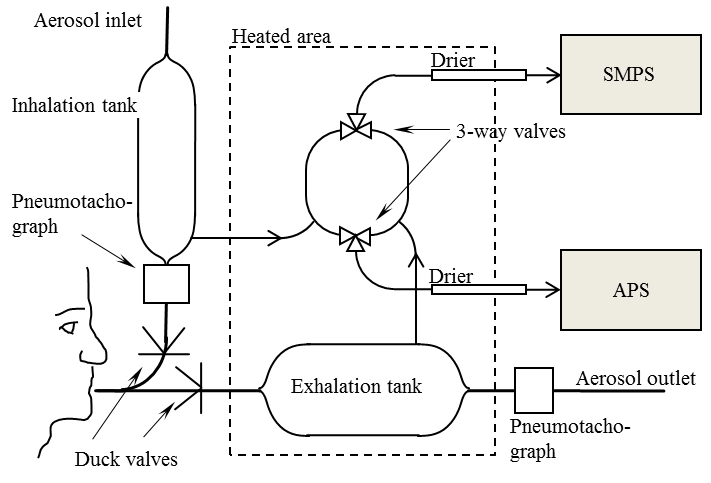
**

**Figure S2.** A schematic drawing of the set-up used for measuring particle lung deposition,

**Table S1.** Results from the bivariate correlation analysis. Coefficients correspond to the Pearson’s correlation coefficients between the independent variables. Significances at the 0.05 and 0.01 level are indicated by * and **, respectively.

|  | Height | VC | TLC | FEV_1_ | V_E_ | RV | V_daw_ | V_DBohr_ | FRC | R20 | R5 | Fres | AX | V_T_ | T_bc_ | Age |
| --- | --- | --- | --- | --- | --- | --- | --- | --- | --- | --- | --- | --- | --- | --- | --- | --- |
| Height | 1 |  |  |  |  |  |  |  |  |  |  |  |  |  |  |  |
| VC | 0.86^**^ | 1 |  |  |  |  |  |  |  |  |  |  |  |  |  |  |
| TLC | 0.87^**^ | 0.92** | 1 |  |  |  |  |  |  |  |  |  |  |  |  |  |
| FEV_1_ | 0.74^**^ | 0.92** | 0.78** | 1 |  |  |  |  |  |  |  |  |  |  |  |  |
| V_E_ | 0.51^**^ | 0.48** | 0.45** | 0.35** | 1 |  |  |  |  |  |  |  |  |  |  |  |
| RV | 0.36^**^ | 0.19 | 0.55** | -0.03 | 0.14 | 1 |  |  |  |  |  |  |  |  |  |  |
| V_daw_ | 0.48** | 0.38** | 0.48** | 0.28* | 0.19 | 0.44** | 1 |  |  |  |  |  |  |  |  |  |
| V_DBohr_ | 0.47** | 0.39** | 0.47** | 0.28* | 0.17 | 0.40** | 0.90** | 1 |  |  |  |  |  |  |  |  |
| FRC | 0.71** | 0.72** | 0.88* | 0.61** | 0.27* | 0.69** | 0.48** | 0.40** | 1 |  |  |  |  |  |  |  |
| R20 | -0.36** | -0.34** | -0.46** | -0.27^*^ | -0.16 | -0.50** | -0.54** | -0.52** | -0.48** | 1 |  |  |  |  |  |  |
| R5 | -0.39** | -0.45** | -0.50** | -0.41^**^ | -0.17 | -0.37** | -0.41** | -0.32* | -0.55** | 0.84** | 1 |  |  |  |  |  |
| Fres | -0.27* | -0.47** | -0.36** | -0.50^**^ | -0.14 | 0.10 | 0.06 | 0.18 | -0.36** | 0.04 | 0.53** | 1 |  |  |  |  |
| AX | -0.35** | -0.53** | -0.44** | -0.55^**^ | -0.11 | 0.03 | -0.05 | 0.07 | -0.41** | 0.15 | 0.58** | 0.91** | 1 |  |  |  |
| V_T_ | 0.35** | 0.37** | 0.34** | 0.33^*^ | 0.09 | 0.03 | 0.17 | 0.44** | 0.14 | -0.09 | 0.06 | 0.13 | 0.08 | 1 |  |  |
| T_bc_ | 0.04 | 0.08 | 0.06 | 0.11 | -0.42^**^ | -0.06 | 0.05 | 0.30* | -0.03 | 0.02 | 0.16 | 0.19 | 0.14 | 0.85** | 1 |  |
| Age | 0.02 | -0.24 | 0.03 | -0.48** | -0.01 | 0.60** | 0.36** | 0.37** | -0.01 | -0.25 | 0.02 | 0.45** | 0.38** | 0.02 | 0.00 | 1 |

**Table S2.** Resulting coefficients from multiple regression analysis for both adults and children.

|  | **Unstandardized Coefficients** | | | | | **Standardized coefficients** | | | |  |
| --- | --- | --- | --- | --- | --- | --- | --- | --- | --- | --- |
| Size intervals | Const. | T_bc_  [min] | V_Daw_  [L] | R5  [kPa·s·L^-1^] | V_T_  [L] | T_bc_ | V_Daw_ | R5 | V_T_ | R |
| **15-30** | 0.592 | 0.911 | -0.787 | 0.134 | 0.164 | 0.329 | -0.552 | 0.196 | 0.388 | 0.835 |
| **30-50** | 0.405 | 1.110 | -0.730 | 0.213 | 0.199 | 0.339 | -0.440 | 0.266 | 0.397 | 0.815 |
| **50-100** | 0.225 | 1.599 | -0.453 | 0.218 | 0.113 | 0.503 | -0.280 | 0.278 | 0.234 | 0.764 |
| **100-200** | *0.224* | *1.076* | *-0.538* | *0.099* | *0.052* | *0.437* | *-0.430* | *0.164* | *0.139* | *0.681* |
| **200-350** | *0.179* | *0.840* | *-0.324* | *0.084* | *0.034* | *0.452* | *-0.342* | *0.183* | *0.119* | *0.640* |
| **850-1250** | *0.189* | *2.236* | *-0.283* | *0.135* | *-0.024* | *0.761* | *-0.188* | *0.178* | *-0.053* | *0.736* |
| **1300-1900** | 0.230 | 2.044 | -0.327 | 0.228 | 0.077 | 0.628 | -0.196 | 0.272 | 0.154 | 0799 |
| **1900-2700** | 0.380 | 1.330 | -0.642 | 0.271 | 0.176 | 0.394 | -0.370 | 0.310 | 0.340 | 0.821 |
| **2700-3500** | 0.509 | 0.704 | -0.429 | 0.287 | 0.173 | 0.258 | -0.305 | 0.407 | 0.414 | 0.796 |
| **3500-5000** | 0.633 | 0.285 | 0.255 | 0.279 | 0.062 | 0.132 | 0.230 | 0.499 | 0.186 | 0.514 |

The multiple regression analyses include the variables T_bc_, V_T_, V_Daw_ and R5. R is the multiple correlation coefficients. Both unstandardized and standardized coefficients are presented, as in Table 3. Standardized coefficients give an indication of the effect of each independent variable on the dependent variable (by standardizing the variables before running the regression, all variables are on the same scale).


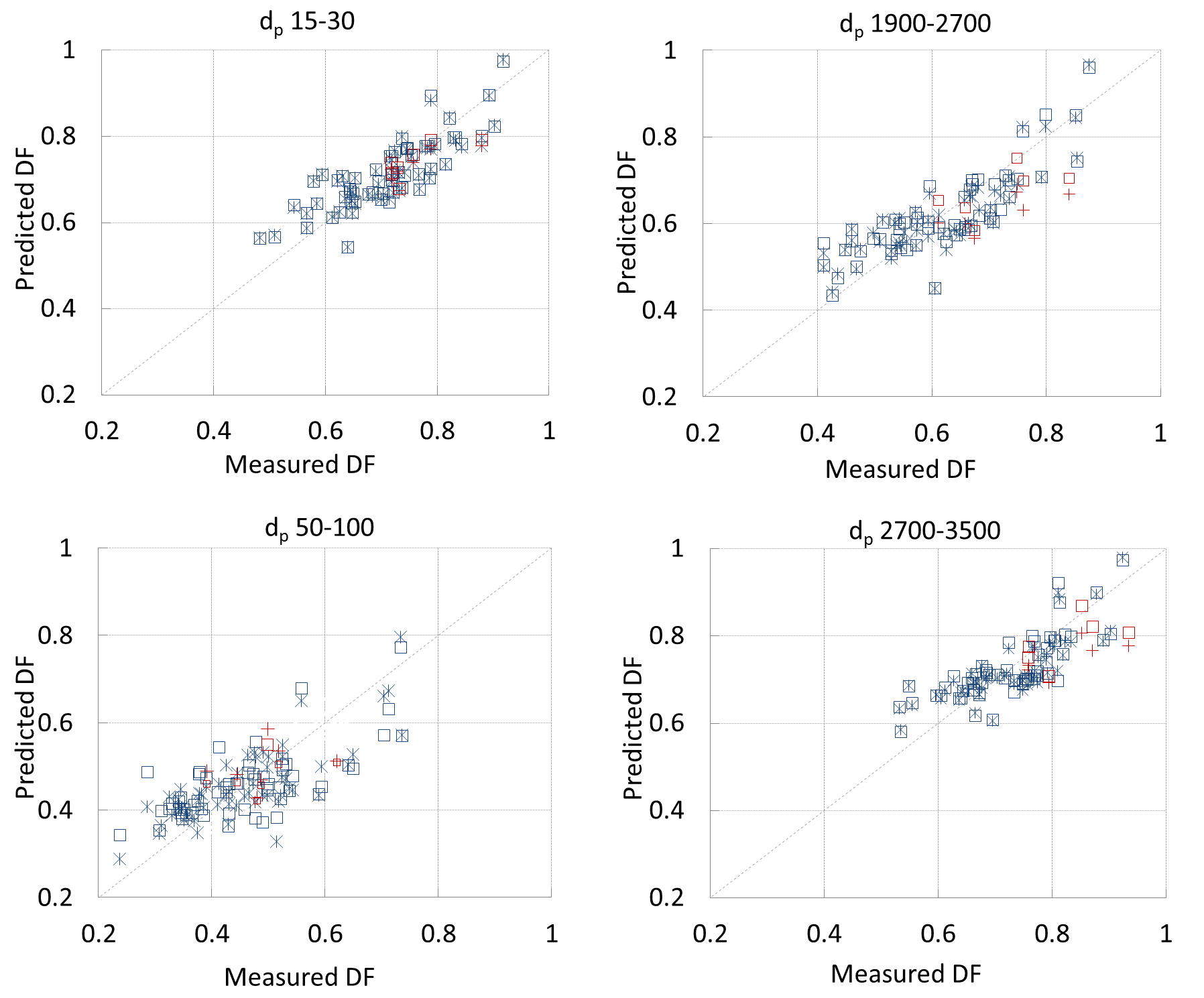


**Figure S3**. The results from a multiple regression analysis including both children and adults. Blue correspond to the adult group and red the children. The results from the first multiple regression analysis including only the adults – as shown in Figure 3 – are also shown (blue **×** for adults and red **+** for children). Including the children improves the level of explanation of the model mainly for the children, not unexpectedly.

**
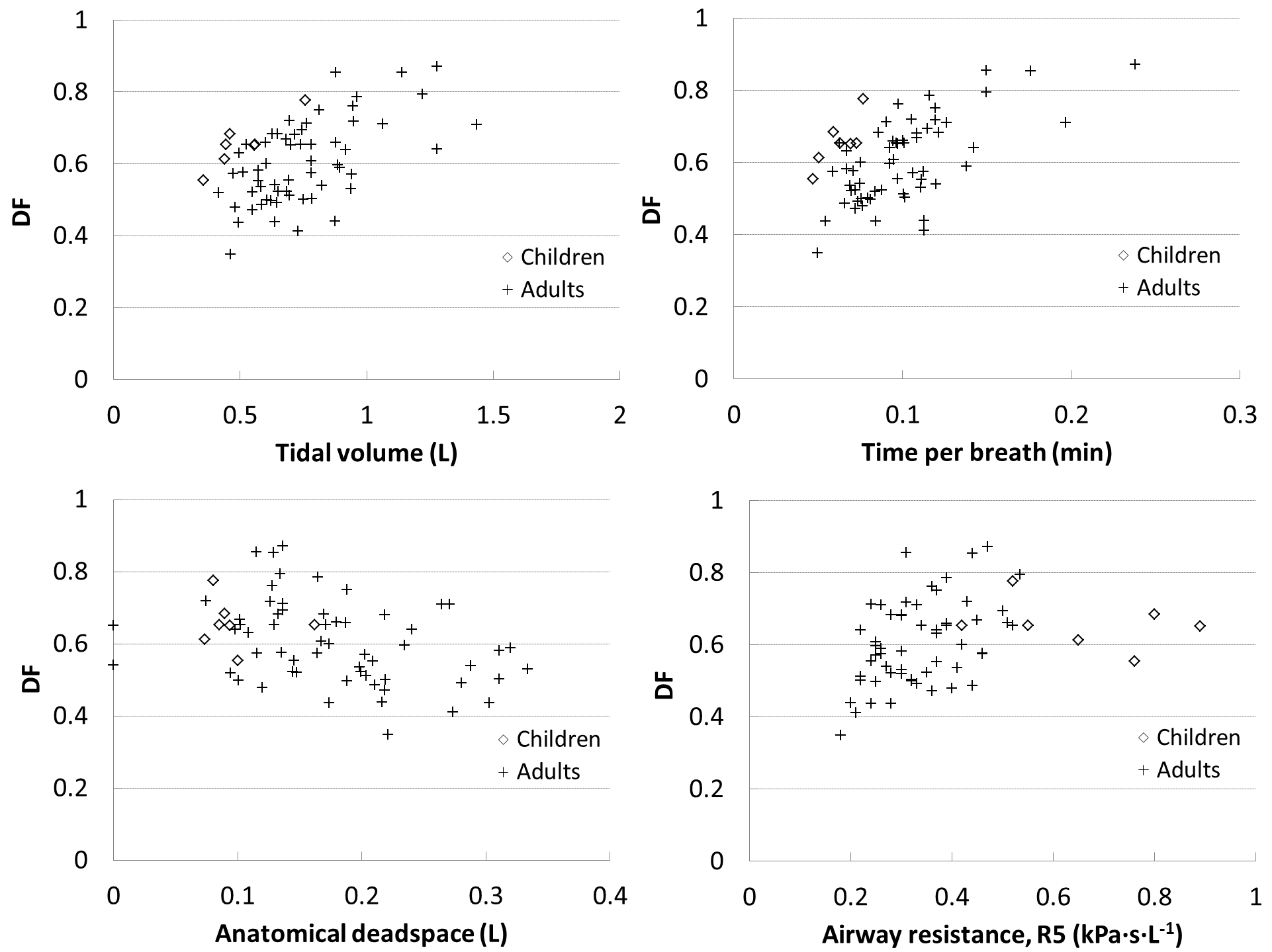
**

**Figure S4.** Adult and children correlation plots.

**Table S3.** Selected results from the lung function investigation and DF (size binned) for each test subject.

|  |  |  |  |  |  |  |  |  |  |  |  |  |  |  |  |  | DF | | | | | | | | | | |
| --- | --- | --- | --- | --- | --- | --- | --- | --- | --- | --- | --- | --- | --- | --- | --- | --- | --- | --- | --- | --- | --- | --- | --- | --- | --- | --- | --- |
|  | Age  y | Length  cm | VC  L | TLC  L | RV  L | FRC  L | FEV1  L | Fres  Hz | AX  kPa/L | R5Hz  kPas/L | R20Hz  kPas/L | V_daw_  L | V_DBohr_  L | T_V_  L | T_bc_  min | V_E_  L/min | 15-  30 | 30-  50 | 50-  100 | 100-  200 | 200-  350 | 800-  900 | 850-  1250 | 1300-  1900 | 1900-  2700 | 2700-  3500 | 3500-  5000 |
| M | 7 | 127 | 1.91 | 2.87 | 1.02 | 1.25 | 1.25 | 16.3 | 1.06 | 0.76 | 0.54 | 0.10 | 0.16 | 0.36 | 0.047 | 7.6 | 0.72 | 0.55 | 0.39 | 0.30 | 0.26 |  |  |  |  |  |  |
| F | 7 | 129 | 2.14 | 2.78 | 0.77 | 1.08 | 1.62 | 20.0 | 2.08 | 0.80 | 0.52 | 0.09 | 0.14 | 0.46 | 0.059 | 7.8 | 0.76 | 0.68 | 0.52 | 0.36 | 0.30 | 0.38 | 0.44 | 0.59 | 0.76 | 0.87 | 0.95 |
| M | 8 | 127 | 2.09 | 2.99 | 0.88 | 1.36 | 1.68 | 15.5 | 0.64 | 0.65 | 0.53 | 0.07 | 0.10 | 0.44 | 0.051 | 8.7 | 0.72 | 0.61 | 0.44 | 0.32 | 0.28 | 0.36 | 0.37 | 0.45 | 0.61 | 0.76 | 0.85 |
| F | 9 | 151 | 2.61 | 3.71 | 1.25 | 1.57 | 1.64 | 25.0 | 2.44 | 0.89 | 0.62 | 0.09 | 0.12 | 0.56 | 0.069 | 8.1 | 0.79 | 0.65 | 0.50 | 0.34 | 0.26 | 0.31 | 0.36 | 0.54 | 0.75 | 0.85 | 0.92 |
| M | 11 | 155 | 3.04 | 4.12 | 1.11 | 1.85 | 2.15 | 20.5 | 1.49 | 0.55 | 0.38 | 0.09 | 0.12 | 0.44 | 0.063 | 7.0 | 0.73 | 0.65 | 0.49 | 0.34 | 0.29 | 0.35 | 0.39 | 0.51 | 0.66 | 0.76 | 0.84 |
| F | 11 | 157 | 3.27 | 4.28 | 0.83 | 1.88 | 2.94 | 10.5 | 0.30 | 0.42 | 0.38 | 0.16 | 0.21 | 0.56 | 0.073 | 7.7 | 0.73 | 0.65 | 0.48 | 0.31 | 0.27 | 0.36 | 0.39 | 0.52 | 0.67 | 0.79 | 0.89 |
| F | 12 | 146 | 2.73 | 3.56 | 0.77 | 1.56 | 2.26 | 12.2 | 0.42 | 0.52 | 0.48 | 0.08 | 0.10 | 0.76 | 0.077 | 9.9 | 0.88 | 0.78 | 0.62 | 0.43 | 0.33 | 0.48 | 0.52 | 0.68 | 0.84 | 0.93 | 0.97 |
| F | 20 | 161 | 3.90 | 4.80 | 0.92 |  | 3.40 |  |  |  |  |  |  | 0.70 | 0.097 | 7.2 | 0.74 | 0.65 | 0.52 | 0.30 | 0.21 | 0.32 | 0.41 | 0.57 | 0.72 | 0.81 | 0.90 |
| F | 20 | 163 | 4.08 | 5.87 | 1.78 | 3.09 | 3.52 | 15.1 | 0.58 | 0.47 | 0.37 | 0.14 | 0.25 | 1.28 | 0.238 | 5.4 | 0.92 | 0.87 | 0.73 | 0.52 | 0.39 | 0.64 | 0.70 | 0.81 | 0.87 | 0.92 | 0.90 |
| F | 20 | 162 | 4.41 | 5.85 | 1.33 | 2.60 | 3.90 | 8.3 | 0.14 | 0.31 | 0.30 | 0.12 | 0.14 | 0.88 | 0.150 | 5.9 | 0.90 | 0.85 | 0.74 | 0.58 | 0.48 | 0.59 | 0.66 | 0.77 | 0.85 | 0.90 | 0.95 |
| M | 21 | 180 | 4.80 | 6.37 | 1.57 | 3.23 | 3.70 | 7.9 | 0.11 | 0.28 | 0.31 | 0.17 | 0.19 | 0.49 | 0.054 | 9.0 | 0.57 | 0.44 | 0.31 | 0.24 | 0.22 | 0.23 | 0.23 | 0.30 | 0.41 | 0.53 | 0.65 |
| M | 22 | 180 | 5.30 | 7.25 | 2.07 | 3.98 | 4.45 | 7.6 | 0.07 | 0.24 | 0.24 | 0.15 | 0.18 | 0.69 | 0.097 | 7.1 | 0.59 | 0.55 | 0.43 | 0.25 | 0.20 |  | 0.33 | 0.45 | 0.51 | 0.72 | 0.81 |
| M | 22 | 180 | 5.72 | 7.37 | 1.73 | 3.83 | 4.85 | 7.9 | 0.08 | 0.25 | 0.26 | 0.17 | 0.19 | 0.78 | 0.095 | 8.2 | 0.63 | 0.61 | 0.50 | 0.33 | 0.25 | 0.34 | 0.38 | 0.49 | 0.59 | 0.69 | 0.78 |
| F | 22 | 168 | 4.64 | 5.53 | 1.09 | 2.31 | 3.76 | 9.8 | 0.21 | 0.37 | 0.32 | 0.10 | 0.13 | 0.92 | 0.092 | 9.9 | 0.83 | 0.64 | 0.42 | 0.28 | 0.23 | 0.32 | 0.36 | 0.51 | 0.68 | 0.80 | 0.91 |
| M | 22 | 171 | 5.37 | 6.94 | 1.46 | 2.95 | 4.86 | 7.6 | 0.08 | 0.24 | 0.25 | 0.14 | 0.18 | 0.76 | 0.091 | 8.4 | 0.79 | 0.71 | 0.59 | 0.46 | 0.38 | 0.44 | 0.48 | 0.59 | 0.70 | 0.79 | 0.91 |
| F | 23 | 161 | 3.68 | 5.47 | 1.75 | 2.90 | 3.12 | 9.1 | 0.20 | 0.30 | 0.28 | 0.09 | 0.13 | 0.42 | 0.084 | 5.0 | 0.65 | 0.52 | 0.34 | 0.26 | 0.20 | 0.26 | 0.27 | 0.35 | 0.46 | 0.55 | 0.62 |
| F | 24 | 164 | 4.11 | 5.43 | 1.32 | 2.61 | 3.30 | 7.3 | 0.08 | 0.44 | 0.48 | 0.21 | 0.23 | 0.59 | 0.066 | 8.9 | 0.54 | 0.49 | 0.35 | 0.19 | 0.16 | 0.23 | 0.26 | 0.35 | 0.41 | 0.65 | 0.91 |
| F | 24 | 170 | 4.38 | 5.96 | 1.52 | 2.87 | 3.71 | 8.0 | 0.08 | 0.30 | 0.30 | 0.22 | 0.24 | 0.72 | 0.109 | 6.6 | 0.77 | 0.68 | 0.54 | 0.32 | 0.27 | 0.39 | 0.43 | 0.57 | 0.66 | 0.75 | 0.87 |
| F | 25 | 170 | 4.53 | 5.57 | 1.08 | 2.34 | 3.77 | 8.2 | 0.10 | 0.37 | 0.33 | 0.19 | 0.22 | 0.81 | 0.119 | 6.8 | 0.82 | 0.75 | 0.64 | 0.40 | 0.32 | 0.44 | 0.51 | 0.65 | 0.74 | 0.82 | 0.94 |
| F | 25 | 164 | 3.79 | 5.51 | 1.74 | 2.81 | 3.12 | 10.1 | 0.24 | 0.31 | 0.29 | 0.13 | 0.16 | 0.95 | 0.119 | 7.9 | 0.83 | 0.72 | 0.53 | 0.36 |  | 0.41 | 0.48 | 0.65 | 0.79 | 0.89 | 0.89 |
| M | 26 | 174 | 5.13 | 7.26 | 1.90 | 3.88 | 4.36 | 13.0 | 0.36 | 0.52 | 0.41 | 0.17 | 0.18 | 0.53 | 0.063 | 8.4 | 0.68 | 0.65 | 0.49 | 0.31 | 0.27 | 0.32 | 0.35 | 0.46 | 0.54 | 0.77 | 1.00 |
| F | 26 | 172 | 4.27 | 5.98 | 1.72 | 3.39 | 3.82 | 8.6 | 0.17 | 0.35 | 0.35 | 0.14 | 0.16 | 0.65 | 0.088 | 7.4 | 0.72 | 0.52 | 0.34 | 0.23 | 0.19 | 0.23 | 0.27 | 0.41 | 0.57 | 0.72 | 0.85 |
| F | 28 | 163 | 3.75 | 5.32 | 1.48 | 2.46 | 3.23 | 11.1 | 0.21 | 0.30 | 0.26 | 0.13 | 0.16 | 0.65 | 0.086 | 7.6 | 0.77 | 0.68 | 0.52 | 0.36 |  | 0.42 | 0.47 | 0.58 | 0.71 | 0.77 | 0.82 |
| F | 28 | 175 | 4.72 | 6.66 | 1.98 | 3.60 | 3.77 | 7.3 | 0.06 | 0.28 | 0.32 | 0.15 | 0.17 | 0.55 | 0.070 | 7.9 | 0.68 | 0.52 | 0.35 | 0.26 | 0.24 | 0.29 | 0.33 | 0.43 | 0.57 | 0.67 | 0.77 |
| M | 29 | 186 | 7.06 | 8.91 | 1.86 | 4.60 | 5.48 | 7.7 | 0.05 | 0.26 | 0.25 | 0.27 | 0.38 | 1.06 | 0.126 | 8.4 | 0.79 | 0.71 | 0.52 | 0.31 | 0.23 | 0.35 | 0.42 | 0.58 | 0.70 | 0.79 | 0.87 |
| F | 29 | 175 | 4.74 | 7.28 | 2.60 | 4.26 | 3.60 | 7.9 | 0.09 | 0.26 | 0.25 | 0.12 | 0.14 | 0.47 | 0.059 | 8.1 | 0.70 | 0.57 | 0.43 | 0.34 | 0.25 | 0.28 | 0.31 | 0.40 | 0.53 | 0.64 | 0.75 |
| M | 30 | 190 | 6.38 | 8.54 | 2.10 | 4.91 | 5.18 | 9.1 | 0.12 | 0.32 | 0.25 | 0.31 | 0.31 | 0.78 | 0.101 | 7.7 | 0.61 | 0.50 | 0.44 | 0.25 | 0.22 | 0.22 | 0.27 | 0.38 | 0.45 | 0.64 | 0.88 |
| F | 30 | 166 | 4.25 | 6.04 | 1.67 | 3.12 | 2.81 | 11.6 | 0.32 | 0.45 | 0.40 | 0.10 | 0.14 | 0.68 | 0.109 | 6.3 | 0.80 | 0.67 | 0.49 | 0.36 | 0.31 | 0.38 | 0.41 | 0.53 | 0.67 | 0.77 | 0.84 |
| F | 31 | 164 | 4.45 | 6.42 | 1.92 | 3.70 | 3.86 | 9.0 | 0.13 | 0.34 | 0.27 | 0.15 | 0.18 | 0.51 | 0.075 | 6.8 |  |  | 0.34 | 0.21 | 0.15 | 0.20 | 0.24 | 0.37 | 0.51 | 0.61 | 0.74 |
| M | 32 | 184 | 6.30 | 8.53 | 2.04 | 4.04 | 4.93 | 8.3 | 0.11 | 0.34 | 0.34 | 0.13 | 0.14 | 0.78 | 0.101 | 7.7 | 0.75 | 0.65 | 0.45 | 0.27 | 0.19 | 0.30 | 0.35 | 0.50 | 0.66 | 0.78 | 0.87 |
| F | 33 | 173 | 4.05 | 5.56 | 1.43 | 3.05 | 3.18 | 7.4 | 0.07 | 0.36 | 0.39 | 0.22 | 0.23 | 0.55 | 0.072 | 7.6 | 0.65 | 0.47 | 0.36 | 0.21 | 0.20 | 0.28 | 0.33 | 0.44 | 0.53 | 0.60 | 0.86 |
| F | 33 | 172 | 4.73 | 6.27 | 1.36 | 2.62 | 3.69 | 8.7 | 0.12 | 0.39 | 0.34 | 0.10 | 0.14 | 0.74 | 0.096 | 7.7 | 0.75 | 0.65 | 0.50 | 0.32 | 0.25 | 0.33 | 0.38 | 0.51 | 0.67 | 0.79 | 0.90 |
| F | 33 | 169 | 3.95 | 6.28 | 2.33 | 3.34 | 3.36 | 9.1 | 0.22 | 0.37 | 0.36 | 0.11 | 0.16 | 0.50 | 0.067 | 7.4 | 0.72 | 0.63 | 0.48 | 0.39 | 0.33 | 0.39 | 0.42 | 0.52 | 0.65 | 0.76 | 0.88 |
| M | 34 | 199 | 8.01 | 9.97 | 1.89 | 4.79 | 6.53 | 8.7 | 0.08 | 0.33 | 0.28 | 0.26 | 0.38 | 1.43 | 0.197 | 7.3 | 0.82 | 0.71 | 0.56 | 0.28 | 0.30 | 0.45 | 0.52 | 0.67 | 0.76 | 0.81 | 0.90 |
| M | 34 | 182 | 6.12 | 8.04 | 1.89 | 3.46 | 4.57 | 8.9 | 0.07 | 0.22 | 0.18 | 0.24 | 0.42 | 1.28 | 0.142 | 9.0 | 0.74 | 0.64 | 0.48 | 0.30 | 0.28 | 0.41 | 0.47 | 0.62 | 0.73 | 0.81 | 0.87 |
| M | 35 | 182 | 6.44 | 8.32 | 1.94 | 3.84 | 4.49 | 5.1 | 0.00 | 0.33 | 0.36 | 0.28 | 0.29 | 0.65 | 0.073 | 8.8 | 0.57 | 0.49 | 0.31 | 0.18 | 0.17 | 0.28 | 0.32 | 0.41 | 0.47 | 0.55 | 0.86 |
| F | 35 | 168 | 4.24 | 6.48 | 2.25 | 3.67 | 3.13 | 7.2 | 0.05 | 0.40 | 0.45 | 0.12 | 0.15 | 0.48 | 0.076 | 6.3 | 0.62 | 0.48 | 0.38 | 0.20 | 0.19 | 0.35 | 0.42 | 0.52 | 0.57 | 0.63 | 0.84 |
| M | 38 | 184 | 6.63 | 8.88 | 2.15 | 4.34 | 4.81 | 7.0 | 0.04 | 0.25 | 0.24 | 0.20 | 0.22 | 0.94 | 0.106 | 8.9 | 0.73 | 0.57 | 0.39 | 0.27 | 0.23 | 0.29 | 0.30 | 0.42 | 0.57 | 0.68 | 0.74 |
| F | 39 | 162 | 3.67 | 5.16 | 1.36 | 1.91 | 2.83 | 9.6 | 0.30 | 0.43 | 0.40 | 0.07 | 0.09 | 0.69 | 0.106 | 6.6 | 0.88 | 0.72 | 0.47 | 0.32 | 0.27 |  |  |  |  |  |  |
| F | 40 | 167 | 4.20 | 6.56 | 2.49 | 3.80 | 3.52 | 8.7 | 0.12 | 0.24 | 0.20 | 0.30 | 0.31 | 0.64 | 0.084 | 7.6 | 0.51 | 0.44 | 0.37 | 0.20 | 0.19 | 0.23 | 0.27 | 0.38 | 0.44 | 0.67 | 0.90 |
| M | 40 | 182 | 5.83 | 9.06 | 3.23 | 5.58 | 4.36 | 9.5 | 0.13 | 0.25 | 0.22 | 0.23 | 0.26 | 0.89 | 0.092 | 9.6 | 0.72 | 0.60 | 0.41 | 0.27 | 0.23 | 0.27 | 0.30 | 0.46 | 0.62 | 0.73 | 0.86 |
| M | 43 | 171 | 4.60 | 6.64 | 1.96 | 2.97 | 3.50 | 13.9 | 0.48 | 0.41 | 0.33 | 0.20 | 0.21 | 0.58 | 0.069 | 8.4 | 0.65 | 0.54 | 0.39 | 0.27 | 0.24 | 0.30 | 0.32 | 0.46 | 0.63 | 0.75 | 0.87 |
| F | 43 | 175 | 5.48 | 7.47 | 2.05 | 3.66 | 3.40 | 8.7 | 0.14 | 0.36 | 0.36 | 0.13 | 0.16 | 0.94 | 0.098 | 9.7 | 0.84 | 0.76 | 0.59 | 0.43 | 0.35 | 0.41 | 0.44 | 0.57 | 0.71 | 0.80 | 0.88 |
| M | 44 | 185 | 5.57 | 7.34 | 1.85 | 3.50 | 4.64 | 8.5 | 0.11 | 0.18 | 0.17 | 0.22 | 0.23 | 0.46 | 0.050 | 9.3 | 0.48 | 0.35 | 0.24 | 0.18 | 0.17 | 0.22 | 0.24 | 0.31 | 0.43 | 0.53 | 0.69 |
| F | 47 | 164 | 3.91 | 5.91 | 1.96 | 3.05 | 3.04 | 9.1 | 0.23 | 0.50 | 0.52 | 0.14 | 0.16 | 0.75 | 0.115 | 6.5 | 0.78 | 0.69 | 0.52 | 0.36 | 0.33 | 0.42 | 0.45 | 0.58 | 0.73 | 0.82 | 0.90 |
| F | 50 | 160 | 3.19 | 4.96 | 1.61 | 1.90 | 2.60 | 8.9 | 0.19 | 0.44 | 0.40 | 0.13 | 0.22 | 1.14 | 0.176 | 6.5 | 0.89 | 0.85 | 0.71 | 0.48 | 0.36 | 0.57 | 0.63 | 0.77 | 0.85 | 0.88 | 0.91 |
| M | 50 | 179 | 5.10 | 7.05 | 2.05 | 2.75 | 3.43 | 11.4 | 0.22 | 0.35 | 0.28 | 0.20 | 0.21 | 0.68 | 0.072 | 9.5 | 0.63 | 0.52 | 0.35 | 0.23 | 0.20 | 0.23 | 0.28 | 0.39 | 0.54 | 0.68 | 0.81 |
| F | 50 | 163 | 3.82 | 5.58 | 1.62 | 2.15 | 2.96 | 14.7 | 0.51 | 0.42 | 0.30 | 0.17 | 0.20 | 0.60 | 0.075 | 8.0 | 0.64 | 0.60 | 0.46 | 0.29 | 0.22 | 0.32 | 0.36 | 0.48 | 0.59 | 0.67 | 0.78 |
| F | 50 | 170 | 3.28 | 4.94 | 1.80 | 2.54 | 2.51 | 15.8 | 0.65 | 0.46 | 0.37 | 0.14 | 0.16 | 0.51 | 0.071 | 7.2 | 0.69 | 0.58 | 0.43 | 0.30 | 0.23 | 0.27 | 0.28 | 0.39 | 0.55 | 0.68 | 0.80 |
| M | 51 | 182 | 5.53 | 7.97 | 2.51 | 3.88 | 3.73 | 12.7 | 0.19 | 0.39 | 0.30 | 0.16 | 0.22 | 0.96 | 0.116 | 8.3 | 0.78 | 0.79 | 0.65 | 0.43 | 0.33 | 0.48 | 0.57 | 0.69 | 0.75 | 0.83 | 0.95 |
| M | 51 | 179 | 5.64 | 8.65 | 3.04 | 5.24 | 3.84 | 7.7 | 0.04 | 0.22 | 0.18 | 0.22 | 0.23 | 0.75 | 0.080 | 9.4 | 0.58 | 0.50 | 0.37 | 0.24 | 0.21 | 0.26 | 0.29 | 0.39 | 0.48 | 0.60 | 0.76 |
| F | 51 | 163 | 3.37 | 5.84 | 2.30 | 3.41 | 2.65 | 11.7 | 0.30 | 0.28 | 0.24 | 0.17 | 0.19 | 0.63 | 0.121 | 5.2 | 0.74 | 0.68 | 0.53 | 0.39 | 0.35 | 0.43 | 0.44 | 0.56 | 0.68 | 0.77 | 0.82 |
| M | 56 | 183 | 5.63 | 8.85 | 3.23 | 5.43 | 3.89 | 7.1 | 0.04 | 0.20 | 0.19 | 0.22 | 0.26 | 0.87 | 0.113 | 7.8 | 0.58 | 0.44 | 0.38 | 0.21 | 0.19 | 0.32 | 0.35 | 0.47 | 0.54 | 0.66 | 0.87 |
| F | 58 | 173 | 4.63 | 6.99 | 2.47 | 3.37 | 3.58 | 12.4 | 0.32 | 0.27 | 0.20 | 0.29 | 0.37 | 0.82 | 0.120 | 6.9 | 0.65 | 0.54 | 0.47 | 0.28 | 0.24 | 0.39 | 0.44 | 0.54 | 0.64 | 0.75 | 0.92 |
| M | 58 | 183 | 4.18 | 6.43 | 2.18 | 2.74 | 3.22 | 15.1 | 0.57 | 0.54 | 0.40 | 0.13 | 0.22 | 1.22 | 0.150 | 8.2 | 0.79 | 0.79 | 0.70 | 0.48 | 0.37 | 0.32 | 0.40 | 0.64 | 0.80 | 0.81 | 0.77 |
| F | 58 | 175 | 4.60 | 7.40 | 2.80 |  | 3.30 |  |  |  |  |  |  | 0.64 | 0.075 | 8.5 | 0.72 | 0.54 | 0.35 | 0.23 | 0.20 | 0.31 | 0.33 | 0.47 | 0.61 | 0.73 | 0.85 |
| F | 59 | 166 | 3.65 | 6.16 | 2.43 | 2.82 | 2.55 | 8.4 | 0.10 | 0.32 | 0.31 | 0.10 | 0.15 | 0.61 | 0.076 | 8.0 | 0.69 | 0.50 | 0.33 | 0.25 | 0.23 | 0.23 | 0.27 | 0.38 | 0.54 | 0.69 | 0.82 |
| F | 60 | 159 | 2.65 | 4.58 | 1.96 | 2.64 | 1.93 | 13.6 | 0.50 | 0.37 | 0.30 | 0.21 | 0.22 | 0.57 | 0.111 | 5.2 | 0.64 | 0.55 | 0.47 | 0.27 | 0.21 | 0.37 | 0.44 | 0.58 | 0.67 | 0.77 | 0.93 |
| M | 61 | 177 | 3.51 | 6.37 | 2.84 | 3.71 | 2.47 | 13.9 | 0.45 | 0.26 | 0.18 | 0.32 | 0.34 | 0.89 | 0.138 | 6.5 | 0.71 | 0.59 | 0.41 | 0.28 | 0.23 | 0.32 | 0.37 | 0.52 | 0.66 | 0.75 | 0.84 |
| F | 61 | 173 | 3.60 | 5.53 | 1.83 | 2.38 | 2.68 | 12.1 | 0.30 | 0.46 | 0.40 | 0.16 | 0.20 | 0.78 | 0.112 | 7.0 | 0.72 | 0.57 | 0.46 | 0.27 | 0.26 | 0.33 | 0.37 | 0.50 | 0.60 | 0.72 | 0.91 |
| M | 62 | 174 | 5.45 | 8.06 | 2.73 | 4.43 | 3.73 | 7.3 | 0.03 | 0.21 | 0.19 | 0.27 | 0.31 | 0.73 | 0.113 | 6.5 |  | 0.41 | 0.29 | 0.19 | 0.16 | 0.29 | 0.35 | 0.47 | 0.56 | 0.64 | 0.88 |
| F | 62 | 157 | 3.38 | 5.48 | 2.09 | 2.36 | 2.54 | 14.8 | 0.60 | 0.39 | 0.28 | 0.19 | 0.20 | 0.60 | 0.094 | 6.4 | 0.74 | 0.66 | 0.54 | 0.33 | 0.27 | 0.40 | 0.45 | 0.57 | 0.64 | 0.70 | 0.81 |
| M | 63 | 179 | 4.99 | 7.64 | 2.65 | 3.66 | 3.32 | 16.0 | 0.41 | 0.30 | 0.19 | 0.33 | 0.43 | 0.94 | 0.111 | 8.5 | 0.63 | 0.53 | 0.38 | 0.25 | 0.27 | 0.33 | 0.35 | 0.44 | 0.46 | 0.66 | 0.86 |
| F | 64 | 164 | 2.78 | 5.06 | 2.23 | 2.37 | 1.72 | 19.8 | 1.53 | 0.51 | 0.31 | 0.18 | 0.29 | 0.88 | 0.100 | 8.7 | 0.72 | 0.66 | 0.50 | 0.31 | 0.24 | 0.35 | 0.43 | 0.59 | 0.67 | 0.76 | 0.82 |
| F | 64 | 172 | 4.08 | 6.20 | 2.19 | 3.51 | 2.89 | 7.0 | 0.05 | 0.25 | 0.26 | 0.19 | 0.20 | 0.62 | 0.081 | 7.7 | 0.70 | 0.50 | 0.33 | 0.26 | 0.24 | 0.32 | 0.34 | 0.43 | 0.55 | 0.67 | 0.73 |
| M | 65 | 176 | 4.69 | 7.10 | 2.43 | 3.78 | 3.52 | 13.1 | 0.35 | 0.30 | 0.22 | 0.31 | 0.38 | 0.57 | 0.067 | 8.5 | 0.64 | 0.58 | 0.51 | 0.41 | 0.33 | 0.42 | 0.51 | 0.60 | 0.61 | 0.70 | 0.89 |
| M | 67 | 175 | 4.89 | 6.80 | 2.21 | 3.29 | 3.25 | 8.4 | 0.06 | 0.22 | 0.19 | 0.20 | 0.27 | 0.70 | 0.101 | 6.9 | 0.65 | 0.51 | 0.43 | 0.26 | 0.25 | 0.32 | 0.35 | 0.45 | 0.50 | 0.66 | 0.91 |

**S3 Particle losses in a tube**

To further investigate the effects of airway dead space on the DF, particle losses (by diffusion and sedimentation) in a horizontal tube were calculated. The calculations were made using the freeware by Paul Baron (Aerosol Calculator), based on the equations in Baron and Willeke [9] and from Hinds [10]. Calculations were performed assuming laminar or turbulent flow.

The following scenarios were evaluated:

1. a decrease in the airway dead space is due to a decrease in all dimensions of the airways, and
2. a decrease in the airway dead space is due to a decrease in airway diameters only.

All calculations were made for the same volume airflow for both scenarios. The constant air volume flow results in higher air velocities and thus shorter particle residence times decreasing the dimensions of the airway dead space. Consequently, a reduced volume of the airways will result in two competing effects: increased particle deposition due to the reduction in the radius of the airway diameters, and decreased deposition due to the reduced residence time. Calculations showed that reducing the dimensions of a tube results in a lower or similar particle deposition, with the exception of the case of diffusion losses for a turbulent flow. In that case, the deposition showed a slight increase. However, it is not likely that the flows were purely turbulent in the lower respiratory tract. Thus, the observed effect of a decreasing DF with increasing V_Daw_ cannot be explained by the deposition in the conducting airways.

**References**

1. Miller MR, Hankinson J, Brusasco V, Burgos F, Casaburi R, Coates A et al. Standardisation of spirometry. Eur Respir J. 2005;26(2):319-38. doi:10.1183/09031936.05.00034805.

2. Wanger J, Clausen JL, Coates A, Pedersen OF, Brusasco V, Burgos F et al. Standardisation of the measurement of lung volumes. Eur Respir J. 2005;26(3):511-22. doi:10.1183/09031936.05.00035005.

3. MacIntyre N, Crapo RO, Viegi G, Johnson DC, van der Grinten CPM, Brusasco V et al. Standardisation of the single-breath determination of carbon monoxide uptake in the lung. European Respiratory Journal. 2005;26(4):720-35. doi:Doi 10.1183/09031936.05.00034905.

4. Brashier B, Salvi S. Measuring lung function using sound waves: role of the forced oscillation technique and impulse oscillometry system. Breathe. 2015;11(1):57-65. doi:10.1183/20734735.020514.

5. Kaminsky DA. What does airway resistance tell us about lung function? Respiratory Care. 2012;57(1):85-99. doi:10.4187/respcare.01411.

6. Wolff G, Brunner JX. Series dead space volume assessed as the mean value of a distribution function. International Journal of Clinical Monitoring and Computing. 1984;1(3):177-81. doi:10.1007/BF01872769.

7. Fletcher R, Jonson B, Cumming G, Brew J. The concept of deadspace with special reference to the single breath test for carbon dioxide. British Journal of Anaesthesia. 1981;53(1):77-88. doi:10.1093/bja/53.1.77.

8. Kars AH, Bogaard JM, Stijnen T, de Vries J, Verbraak AF, Hilvering C. Dead space and slope indices from the expiratory carbon dioxide tension-volume curve. Eur Respir J. 1997;10(8):1829-36.

9. Baron A, Willeke K. Aerosol Measurement: Principles, Techniques and Applications. New York: John Wiley and Sons, Inc.; 2005.

10. Hinds. Aerosol Technology. 1982.
